# Differences in Mortality Between Treatment-Naive and Treatment-Discontinuing Hospitalized Individuals With Advanced HIV Disease: A Comparative Retrospective Study From Mexico City

**DOI:** 10.1093/cid/ciaf175

**Published:** 2025-04-03

**Authors:** Héctor Rivera-Villegas, Yanink Caro-Vega, André Trujillo-Gamboa, Álvaro Lopez-Iñiguez, Juan Sierra-Madero, Brenda Crabtree-Ramirez

**Affiliations:** Infectious Diseases Department, Instituto Nacional de Ciencias Médicas y Nutrición Salvador Zubirán, Mexico City, Mexico; Infectious Diseases Department, Instituto Nacional de Ciencias Médicas y Nutrición Salvador Zubirán, Mexico City, Mexico; Infectious Diseases Department, Instituto Nacional de Ciencias Médicas y Nutrición Salvador Zubirán, Mexico City, Mexico; Infectious Diseases Department, Instituto Nacional de Ciencias Médicas y Nutrición Salvador Zubirán, Mexico City, Mexico; Infectious Diseases Department, Instituto Nacional de Ciencias Médicas y Nutrición Salvador Zubirán, Mexico City, Mexico; Infectious Diseases Department, Instituto Nacional de Ciencias Médicas y Nutrición Salvador Zubirán, Mexico City, Mexico

**Keywords:** HIV, mortality, treatment-abandoned, advanced HIV disease, antiretroviral treatment

## Abstract

**Background:**

Advanced human immunodeficiency virus (HIV) disease is a major cause of morbidity and mortality in people with HIV in Latin America. It remains unclear whether mortality differs between people with advanced HIV who are treatment naive (TN) and those who have discontinued antiretroviral treatment (TD). We compared mortality differences between hospitalized TN and TD individuals with advanced HIV.

**Methods:**

All adults hospitalized for advanced HIV disease (CD4 count <200 cells/μL or with AIDS-defining events) at a tertiary center in Mexico City between January 2015 and December 2022 were included. The primary outcome was 1-year mortality following hospitalization. Secondary outcomes were overall mortality and 30-day mortality.

**Results:**

Four hundred seventy hospitalizations occurred in 299 adults with advanced HIV. Of these, 214 (72%) were TN at the time of admission, while 85 (28%) were TD. The median CD4 cell count was 76 cells/μL and AIDS-related infections accounted for most of the deaths. No differences were found between the groups regarding hospitalization days, CD4 cell count, or comorbidities. TD were more likely to have acquired HIV by sexual transmission (*P* = .009). One-year mortality was significantly higher in the TD group (24% vs 8%; *P* = .008). The overall mortality was also higher among TD individuals (*P* = .008). Multivariate analysis revealed an independent association between treatment discontinuation and mortality (hazard ratio, 2.08 [95% confidence interval, 1.14–3.78]).

**Conclusions:**

Treatment discontinuation is associated with worse clinical outcomes when compared to TN patients. Future research should focus on understanding the reasons for these findings to develop public health strategies to prevent disruption of the continuum of care.

## Teaser

Advanced HIV disease in Latin America remains a public health problem, affecting treatment-naive (TN) individuals and those who discontinue treatment (TD). We compared differences in mortality between TN and TD individuals, finding higher mortality rates in TD (24%) versus TN (8%).

## Graphical Abstract

**Graphical Abstract:**
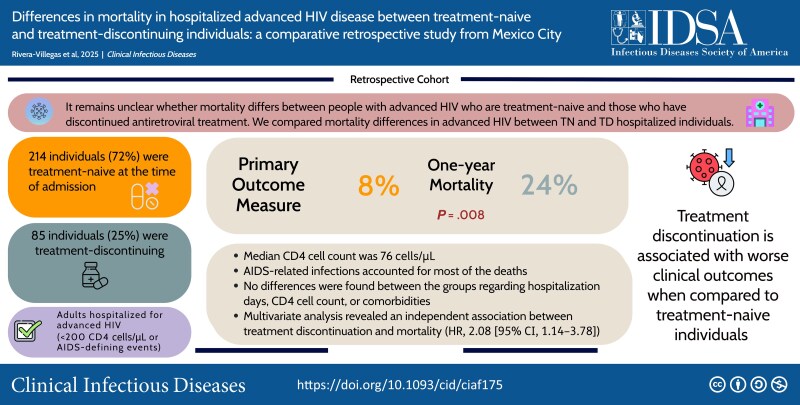
This graphical abstract is also available at Tidbit: https://tidbitapp.io/institutional-portal/clinical-infectious-diseases/tidbits/differences-in-mortality-in-hospitalized-advanced-hiv-disease-between-treatment-naive-and-treatment-discontinuing-individuals-a-comparative-retrospective-study-from-mexico-city/update

The care of patients with advanced human immunodeficiency virus (HIV) disease remains a significant challenge for global health, affecting approximately 11%–60% of newly diagnosed people with HIV (PWH) [1, 2]. It is estimated that around 4 million people have advanced HIV disease, resulting in >600 000 deaths annually. This issue is particularly relevant in developing countries, where the needs of PWH differ from the priorities of public health programs, patient care, and research in developed nations. Despite a decrease in inequality in HIV care over the past decades, low- and middle-income countries contribute to most of the HIV mortality burden. Although efforts to improve access to HIV screening have been made, many barriers persist that limit early detection.

Despite a decline in AIDS-related deaths [3], mortality associated with advanced HIV disease remains high worldwide and continues to contribute to most HIV-related hospital admissions [4], particularly in developing countries. In this regions, weak and fragmented health systems prevail, as well as discrimination, stigma, forced migration, high transportation costs, and social determinants, which remain a major challenge [5].

Latin America is 1 of the few regions of the world in which new HIV cases are rising [6]. Additionally, the coronavirus disease 2019 pandemic has jeopardized the continuum of HIV care in many settings [7, 8]. Consequently, not only has a lack of access to diagnosis been described, but also antiretroviral therapy (ART) discontinuation. The latter poses a significant challenge in HIV management, potentially accelerating disease progression and heightening the risk of severe complications, which can lead to an increased frequency of hospitalizations due to advanced disease. Several factors associated with treatment interruption have been identified, including substance use, adverse treatment effects, and lack of social support, among others [9]. In Mexico, the complex and fragmented healthcare system presents a considerable challenge for efficient transfer between different care systems, which involves a great burden of bureaucracy, logistics, and time, which can potentially contribute to interruptions of the HIV continuum of care for our patients [10].

Several demographic factors can contribute to late diagnosis and advanced HIV disease. These factors include belonging to marginalized groups, limited access to diagnostic tests, older age, and long distances to health centers [11]. Additionally, social factors such as stigma, low risk perception, drug use, and unemployment contribute to a late diagnosis [12, 13]. Some of these factors, such as drug use and older age, are also associated with loss to follow-up and treatment discontinuation [14].

The fragmentation of healthcare systems and the transition between different facilities potentially increase treatment discontinuation, although their impact is not yet well understood [10, 15]. While the effect of advanced HIV disease on quality of life and mortality is well documented [16], it remains unclear whether mortality rates and adverse outcomes differ between individuals experiencing AIDS-defining events who require hospitalization but have not yet initiated ART, compared to those with advanced disease resulting from treatment discontinuation.

Therefore, we aim to assess mortality rates between treatment-naive and treatment-discontinuing individuals in people with advanced HIV disease who require hospitalization.

## METHODS

We conducted a retrospective cohort study at a tertiary care referral center located in Mexico City, Mexico, which provides care to a population of >2500 PWH. We collected data from electronic medical records of all patients hospitalized between January 2015 and December 2022 due to advanced HIV infection. We established 2015 as the start year of data collection because it marked the beginning of universal access to ART in Mexico, regardless of CD4 cell count.

We included individuals aged ≥18 years who were hospitalized for advanced HIV disease–related complications. Advanced HIV disease was defined as having a CD4 count <200 cells/μL and/or the presence of AIDS-defining illness, in accordance with World Health Organization guidelines [17]. We classified treatment-naive individuals as those with advanced HIV disease who have never taken ART, while treatment-discontinuing individuals were defined as those with a history of ART initiation and loss to follow-up with ART discontinuation for >6 months. We collected information regarding demographic, clinical, and microbiological aspects; total days and causes of hospitalization; and mortality data (death and causes of death) from the electronic medical records and from the HIV Clinic database. The presence of comorbidities was defined as having diabetes, non-AIDS-defining cancer, or cardiovascular disease. We excluded individuals with incomplete data and those with hospital admissions unrelated to advanced HIV disease.

The primary outcome was defined as 1-year mortality following the first hospital admission due to advanced HIV disease. Secondary outcomes included 30-day mortality after hospital admission, overall mortality (death due to any cause during the follow-up period), and the duration of hospitalization. Advanced HIV–related deaths were categorized as either infection related or cancer related. A coded and encrypted database, accessible solely to the researchers, was employed for data storage and analysis.

We calculated a sample size of 252 individuals, to estimate a 20% difference in mortality during the first year after the date of hospitalization between the treatment-naive group and the treatment-discontinuation group, along with a mean absolute percentage error of 10% and a group distribution ratio of 1:3, reflecting the ratio of individuals who discontinue treatment to those who were treatment naive [18].

Baseline clinical and demographic characteristics were described based on data recorded at the first hospital admission, using measures of central tendency: mean with standard deviation (SD) for normally distributed quantitative variables, and median with interquartile range (IQR) for nonnormally distributed variables. Continuous variables were assessed using mean, median, SD, and range. We estimated the frequency and percentage of deaths at 1 year and 30 days after the hospitalization date. A Cox proportional hazards model was performed to assess factors associated with mortality within the first year of hospital admission and to plot adjusted 1-year survival curves by group. The model included the following covariates: sex at birth, age at first hospitalization event (treated as a continuous variable using splines), HIV sexual acquisition risk, number of hospitalization events (categorized as 1 or ≥2), and presence of comorbidities. We used multiple imputation with 5 replications to address missing covariates. The model was constructed using covariates of biological importance and clinical relevance. Proportions were compared using χ^2^ or Student *t* test, with statistical significance set at *P* < .05. Statistical analyses were conducted using R software (version 4.3.1).

Because of the study's retrospective nature, the informed consent requirement was waived. The study, including the waived informed consent, was approved by the Review Board of Instituto Nacional de Ciencias Médicas y Nutrición Salvador Zubirán (reference number 4602). All personal data were protected according to national and international standards.

## RESULTS

### Study Population

**Figure 1.**
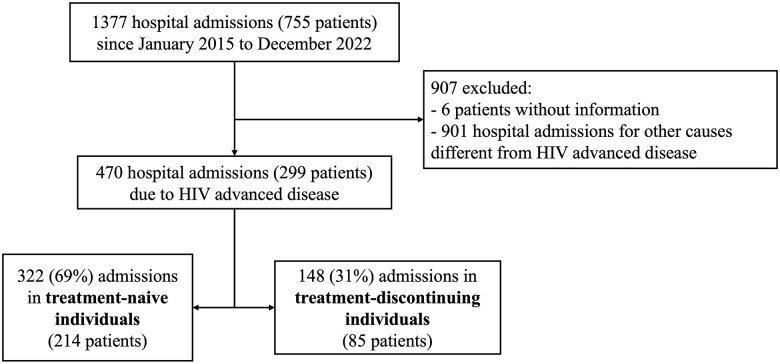
Flow diagram of patient inclusion. Abbreviation: HIV, human immunodeficiency virus.

The distribution of patients is shown in Figure 1. During the study period, a total of 1377 hospital admissions of PWH were recorded, corresponding to 755 individuals. Of these, 901 admissions, corresponding to 456 patients, were excluded from the analysis due to non–advanced HIV–related hospitalization. Additionally, 6 patients were excluded due to insufficient information in their clinical records. Ultimately, we included 299 patients in the analysis, of whom 214 (72%) admissions were in treatment-naive individuals and 85 (28%) were among those who discontinued ART.

Overall, 261 of 299 (87%) individuals were male, with a median age of 37 years and a median of 13 years of education. Sexual transmission accounted for most HIV acquisition risk, occurring in 264 (88%) individuals. Forty-three of the included individuals had comorbidities (diabetes, hypertension, cardiovascular disease, or cancer), and the overall median CD4 cell count was 76 cells/μL. When comparing the 2 groups, only sexual transmission as a mode of HIV acquisition was significantly more common in the treatment-naive group (*P* = .009) (Table 1).

### Clinical Outcomes

Within 1 year of hospitalization, mortality was observed in 47 individuals (16%) and was significantly more frequent in the group that discontinued treatment (27%), compared to the treatment-naive group (11%) (*P* = .008; Table 2). When examining the overall mortality, a statistically significant difference was found between the treatment-discontinuation group and the treatment-naive group (27% vs 12%, respectively; *P* = .008). Although the treatment-discontinuation group experienced a higher number of deaths within 30 days of admission (24% vs 8%), this difference was not statistically significant (*P* = .062). The global median number of hospitalization days was 10 (IQR, 5–18). Most deaths occurred during hospitalization (36/47 cases [77%]). Among individuals who died after discharge from the hospital, the median number days of survival was 11 (IQR, 31–63). No statistically significant differences were observed between the cause of death (infectious or neoplastic), admission diagnosis, and hospitalization days between the 2 groups (Table 2). In the treatment-naive group, 73 (34%) were diagnosed with HIV during hospitalization, and the median number of days from diagnosis to hospitalization was 14 (IQR, 1–55).

**Table 1.** Demographic and Clinical Characteristics at First Hospitalization

Variable	Total (n = 299)	Treatment Naive (n = 214 [72%])	Treatment Discontinued (n = 85 [28%])	*P* Value
Male sex, No. (%)	261 (87)	189 (88)	72 (85)	.513
Age, y, median (IQR)	37 (30–45)	36 (30–46.75)	37 (30–44)	.807
Education, y, median (IQR)	13 (9–15)	14 (9–15)	12 (9–12)	.079
HIV sexual transmission^a^, No. (%)	264 (88)	191 (90)	73 (86)	.009
Comorbidities^b^, No. (%)	90 (30)	59 (28)	31 (36)	.169
Hospitalization days^c^, median (IQR)	10 (5–18)	10 (5–14.6)	9 (4–13.6)	.502
CD4 count^d^, cells/μL, median (IQR)	44 (16–106)	46.5 (17–106)	40 (14–106)	.735

Abbreviations: HIV, human immunodeficiency virus; IQR, interquartile range.

^a^Sexual transmission was categorized as sexual vs others forms of transmission.

^b^Comorbidities included the diagnoses of diabetes, non-AIDS-defining cancers, or cardiovascular disease.

^c^Hospitalization days were calculated based on the first hospitalization due to advanced HIV disease.

^d^Seventeen percent of CD4 cell counts were missing and were corrected using multiple imputation.

**Table 2.** Mortality Outcomes

Variable	Total (n = 299)	Treatment Naive (n = 214 [72%])	Treatment Discontinued (n = 85 [28%])	*P* Value
1-y mortality	47 (16)	24 (11)	23 (27)	.008
Overall mortality	48 (16)	25 (12)	23 (27)	.008
30-d mortality	38 (13)	18 (8)	20 (24)	.062
Cause of death				
Infections	33 (69)	71	16	.504
Cancer	15 (31)	10	5	

Data are presented as No. (%) unless otherwise indicated.

**Figure 2.**
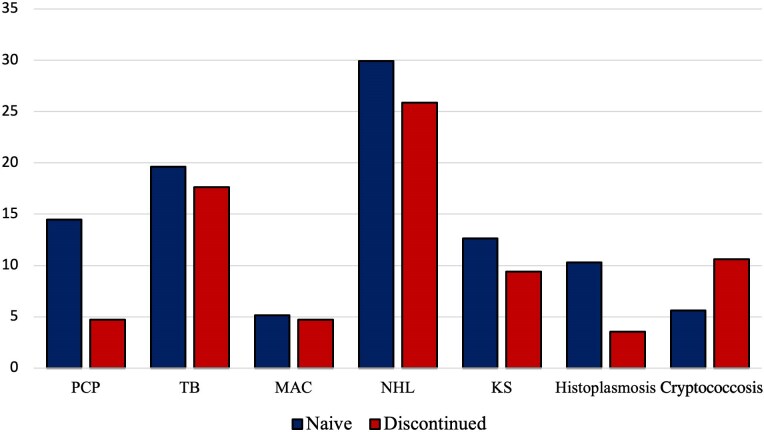
Percentage of AIDS-defining events at hospitalization. Abbreviations: KS, Kaposi sarcoma; MAC, *Mycobacterium avium* complex infection; NHL, non-Hodgkin lymphoma; PCP, *Pneumocystis jirovecii* pneumonia; TB, tuberculosis.

AIDS-related infections accounted for most of the deaths, which occurred in 33 individuals (69%), with pneumonia being the most prevalent cause. However, non-Hodgkin lymphoma was the AIDS-defining disease with the highest number of deaths. The frequency of admission diagnoses was as follows: non-Hodgkin lymphoma (n = 86 [29%]), tuberculosis (n = 57 [19%]), *Pneumocystis jirovecii* infection (n = 35 [18%]), histoplasmosis (n = 25 [13%]), and cryptococcosis (n = 21 [7%]). The percentages of AIDS-defining events at hospitalization are shown in Figure 2.

### Factors Associated With Death

**Figure 3.**
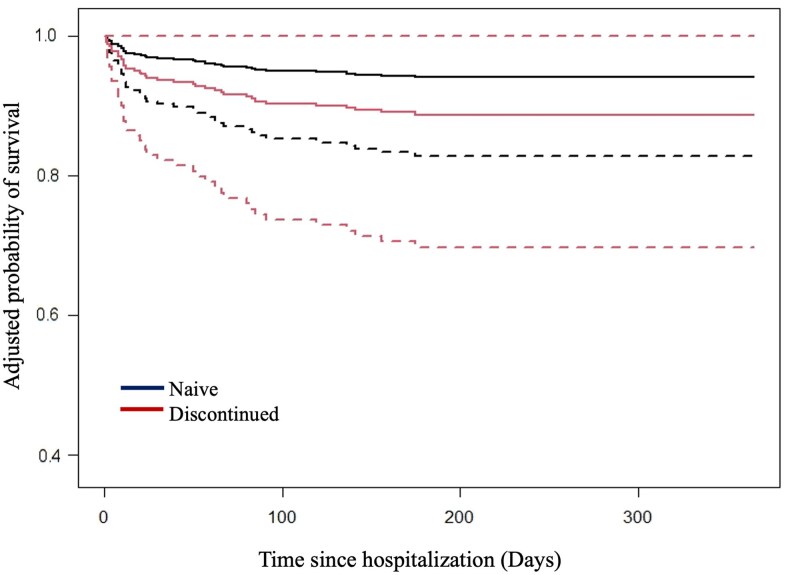
One-year adjusted survival according to treatment-naive and treatment-discontinued groups. Predicted survival curves are for 45-year-old men with comorbidities reported, who had a CD4^+^ count of 50 cells/μL, human immunodeficiency virus acquisition risk of sexual contact, and 2 or more hospitalization events.

In a multivariate analysis adjusting for sex, age, comorbidities, sexual transmission, CD4 cell count, and number of hospitalizations, treatment discontinuation (defined as history of loss to follow-up and ART discontinuation for >6 months) was the only variable that demonstrated an independent association with 1-year mortality (hazard ratio, 2.08 [95% confidence interval {CI}, 1.14–3.78]) (Table 3). The 1-year adjusted survival curves for the treatment-naive and treatment-discontinuation groups are shown in Figure 3.

**Table 3.** Multivariate Analysis for Primary Outcome

Variable	aHR (95% CI)	*P* Value
Treatment group		
Naive	1	.016
Discontinued	2.08 (1.14–3.78)	
Sex at birth		
Male	1	.832
Female	0.90 (.13–2.32)	
Age, y		
25	1	.38
45	1.79 (.96–4.63)	
50	1.83 (.74–4.53)	
CD4 count, cells/μL		
50	1	.86
200	0.70 (.35–1.38)	
350	0.49 (.12–1.91)	
HIV acquisition risk		
Nonsexual	1	.62
Sexual	0.77 (.27–2.19)	
Comorbidities		
No	1	.99
Yes	1.00 (.55–1.82)	
No. of hospitalizations		
1	1	.30
≥2	0.34 (.27–2.18)	

Abbreviations: aHR, adjusted hazard ratio; CI, confidence interval; HIV, human immunodeficiency virus.

## DISCUSSION

We found a higher percentage of 1-year mortality among those who discontinued ART in the adjusted model, with opportunistic infections being the main cause of death in the analyzed population.

Our study highlights a significant problem in our region: the late diagnosis of HIV, which contributes substantially to HIV-related mortality, despite universal access to ART for >2 decades. This is reflected in our study by the significantly low CD4 counts observed among hospitalized individuals (median, 76 cells/μL) and the high mortality rate due to AIDS-related infections. In contrast to the 30% of HIV-related deaths in developed countries in PWH [19, 20], the burden of advanced HIV disease in our region is unacceptably high. Approximately 80% of HIV-associated deaths could be prevented if advanced HIV disease is avoided [21]. This underscores the urgent need for more effective health policies to increase HIV testing, linkage to care, retention, and prevention of loss to follow-up. Additionally, there is limited evidence of successful interventions that reduce treatment interruptions and improve adherence to the healthcare system in our context [22].

In the present study, the difference in mortality between treatment-naive patients with advanced disease and those who had discontinued treatment is striking. Mortality among treatment-naive individuals with advanced HIV disease has been reported to range from 3% to 20% [23–25], which is similar to the proportion found in our study. Several studies have confirmed that treatment discontinuation leads to a higher risk of all-cause mortality, an increased incidence of opportunistic infections, and a lower quality of life [26]. However, there are no studies specifically comparing advanced disease outcomes between these 2 groups. This finding underscores the importance of establishing mechanisms and standardizing procedures to effectively trace patients lost to follow-up and to identify structural deficiencies within the healthcare system that may jeopardize the continuity of care for PWH.

Antiretroviral treatment discontinuation increases the risk of death, opportunistic infections, and poor immune recovery. Compared to a previous study conducted in our region, in which 11% of admissions were related to treatment discontinuation [27], the rate of advanced HIV disease hospitalizations due to treatment discontinuation has risen markedly. In our study such cases account for almost one-third of patients admitted to our institution, a tertiary referral center. A global rate of 20% for treatment discontinuation has been described, with the main risk factors being adverse effects, drug toxicity, and intravenous drug use [14]. A systematic review conducted by Burke et al on the reasons for treatment abandonment demonstrated the complex nature of this phenomenon, with reasons as varied as substance use, lack of social and psychological support, caregiving and social responsibilities, and unexpected mobility [9]. However, not all treatment discontinuations result in the development of advanced HIV disease, and the factors associated with HIV disease progression in this subgroup of patients remain unknown and require further investigation. Additionally, many studies suggest that HIV-related stigma plays a key role on delaying access to care and ART initiation, as it can lead to a lower use of healthcare services, which may negatively impact appropriate adherence to ART and result in unfavorable outcomes [28]. Therefore, it is crucial to understand how social factors contribute to disease progression in patients who discontinue HIV treatment and whether these factors contribute to excess mortality in this subgroup.

The proportion of advanced HIV disease–related mortality found in our study falls within the range reported in other countries in our region and in other developing countries [25, 29]. In a study from Malawi, 20% advanced HIV disease–related mortality, primarily due to tuberculosis, was described [29]. As with other cohorts in our region, opportunistic infections are the primary cause of hospitalization in PWH. In a European and North American cohort, the relative risk of mortality was found to be higher among people with HIV when restarting treatment after an interruption than among people starting ART for the first time; the adjusted hazard ratio for the group after an interruption was 1.72 (95% CI, 1.57–1.88) [30]. Another French cohort found that interruption of medical care led to a significant decrease in CD4 levels and was independently associated with the development of AIDS (odds ratio [OR], 2.54 [95% CI, 2.10–3.09]) and death (OR, 2.65 [95% CI, 1.94–3.61]) [31].

Overall, non-Hodgkin lymphoma was the most common AIDS-defining condition leading to death. While AIDS-associated infections accounted for most fatalities, individuals with lymphoma exhibited the highest mortality rates. This contrasts with other studies on mortality in advanced HIV disease, where tuberculosis, cryptococcal meningitis, and severe bacterial infections (bacteremia, pneumonia, and diarrhea) have been described as the main causes of death [29, 32]. It is unknown whether this finding could be related to the type of institution where the study was conducted, as hematological pathologies may be overrepresented. Interestingly, no differences were found in the CD4 cell counts or the types of opportunistic diseases between those who died in the 2 groups. However, a significant difference was observed in the HIV transmission risk in those who had discontinued treatment, with parenteral transmission associated with intravenous drug use predominating in our country. Intravenous drug use is a commonly reported risk factor for ART discontinuation [14], which may explain the higher frequency of parenteral transmission in the discontinuation group.

Treatment discontinuation is a current and significant problem in the HIV population. Patients who discontinue treatment are more likely to suffer from adverse circumstances that may contribute to poorer clinical outcomes, such as lack of social security, intravenous drug use, and long-term HIV infection [33]. The role of stigma and its impact on treatment adherence should not be underestimated. Healthcare system fragmentation in our country could also significantly contribute to ART discontinuation due to the lack of continuity between different healthcare providers [10].

It remains unclear whether social determinants, such as food insecurity, homelessness, violence, and unemployment contribute to a poorer health status and increase mortality, or if these social determinants primary act as barriers to continuity of care, leading to loss to follow-up and ART discontinuation, which may explain the higher mortality observed compared to those with advanced HIV disease due to late diagnosis. More research is needed to determine whether these unfavorable social conditions are more pronounced in patients who discontinue treatment than in those who are diagnosed late, as this may impact the differences in mortality between the groups. Regarding possible biological factors that could explain these findings, Thomadakis et al reported a slower recovery of CD4 cell count upon resumption of ART after a period of discontinuation [34]. This could lead to a higher risk of death and advanced HIV disease. The fact that no differences in CD4 counts were observed in our study suggests that immunological/biological factors beyond absolute CD4 count may contribute to these important differences, warranting further investigation.

Our study has several limitations. The retrospective nature should be considered; however, information was systematically collected and very few data were missing. The reasons for treatment abandonment were not recorded. The single-center nature of our study may limit the reproducibility of these results in a region with unequal access to diagnostic and therapeutic resources. In addition, other biological, immunological, and sociodemographic variables were not examined, which could provide a more comprehensive understanding of these findings. Nonetheless, the sample size and extensive data collection are strengths of our study. Given the significant differences observed between the groups, this research warrants deserves attention, particularly in a region in which advanced HIV disease remains prevalent.

Advanced HIV disease has primarily been viewed and analyzed as a problem associated with late diagnosis, resulting in the implementation of early detection strategies with varying effectiveness in our region. However, advanced disease also affects individuals who have already initiated treatment but have not successfully maintained follow-up and adherence to the healthcare system. In some regions, such as South Africa, disengagement has become the leading cause of morbidity associated with HIV [35]. It is imperative for health policies to also focus on maintaining continuity of care for people with HIV. Such measures could potentially prevent up to one-third of hospitalizations and deaths due to advanced HIV–related illnesses.

## CONCLUSIONS

Advanced HIV disease mortality related to ART discontinuation is higher than among people with advanced disease who are treatment naive. Unidentified biological or social factors may explain these outcomes. Future research should focus on understanding the reasons for these differences and on developing public health strategies to prevent treatment interruptions.

## Notes

***Author Contributions.*** Study conception: B. C.-R., H. R.-V., and Y. C.-V. Data analysis and input parameter development: Y. C.-V., B. C.-R., and H. R.-V. Interpretation of results: All authors. Manuscript drafting: B. C.-R. and H. R.-V. Editing, revisions, and final approval of the submitted version: All authors.

***Financial support.*** This work was supported by the NIH-funded Caribbean, Central and South America Network for HIV Epidemiology (CCASAnet), a member cohort of the International epidemiology Databases to Evaluate AIDS (IeDEA) (grant number U01AI069923). This award is funded by the following institutes of the NIH: the National Institute of Allergy and Infectious Diseases, the Eunice Kennedy Shriver National Institute of Child Health and Human Development, the National Heart, Lung, and Blood Institute, the National Institute of Diabetes and Digestive and Kidney Diseases, the National Institute on Drug Abuse, the National Institute on Alcohol Abuse and Alcoholism, the Fogarty International Center, and the National Cancer Institute.
